# A priori prediction of local failure in brain metastasis after hypo-fractionated stereotactic radiotherapy using quantitative MRI and machine learning

**DOI:** 10.1038/s41598-021-01024-9

**Published:** 2021-11-03

**Authors:** Majid Jaberipour, Hany Soliman, Arjun Sahgal, Ali Sadeghi-Naini

**Affiliations:** 1 Department of Electrical Engineering and Computer Science, Lassonde School of Engineering, York University, Toronto, ON Canada; 2 Physical Sciences Platform, Sunnybrook Research Institute, Sunnybrook Health Sciences Centre, Toronto, ON Canada; 3 Department of Radiation Oncology, Odette Cancer Centre, Sunnybrook Health Sciences Centre, Toronto, ON Canada; 4 Department of Radiation Oncology, University of Toronto, Toronto, ON Canada; 5 Department of Medical Biophysics, University of Toronto, Toronto, ON Canada

**Keywords:** Biomedical engineering, Predictive markers, Prognostic markers, Cancer, Biophysics, Cancer imaging

## Abstract

This study investigated the effectiveness of pre-treatment quantitative MRI and clinical features along with machine learning techniques to predict local failure in patients with brain metastasis treated with hypo-fractionated stereotactic radiation therapy (SRT). The predictive models were developed using the data from 100 patients (141 lesions) and evaluated on an independent test set with data from 20 patients (30 lesions). Quantitative MRI radiomic features were derived from the treatment-planning contrast-enhanced T1w and T2-FLAIR images. A multi-phase feature reduction and selection procedure was applied to construct an optimal quantitative MRI biomarker for predicting therapy outcome. The performance of standard clinical features in therapy outcome prediction was evaluated using a similar procedure. Survival analyses were conducted to compare the long-term outcome of the two patient cohorts (local control/failure) identified based on prediction at pre-treatment, and standard clinical criteria at last patient follow-up after SRT. The developed quantitative MRI biomarker consists of four features with two features quantifying heterogeneity in the edema region, one feature characterizing intra-tumour heterogeneity, and one feature describing tumour morphology. The predictive models with the radiomic and clinical feature sets yielded an AUC of 0.87 and 0.62, respectively on the independent test set. Incorporating radiomic features into the clinical predictive model improved the AUC of the model by up to 16%, relatively. A statistically significant difference was observed in survival of the two patient cohorts identified at pre-treatment using the radiomics-based predictive model, and at post-treatment using the the RANO-BM criteria. Results of this study revealed a good potential for quantitative MRI radiomic features at pre-treatment in predicting local failure in relatively large brain metastases undergoing SRT, and is a step forward towards a precision oncology paradigm for brain metastasis.

## Introduction

Brain metastasis patients still suffer from poor prognosis despite the recent advances in cancer treatment^1,2^. The treatment options for brain metastasis include radiation therapy, surgery, and systemic treatment^3,4^. Radiation therapy may be administrated through whole brain radiation therapy (WBRT), single-fraction stereotactic radiosurgery (SRS), or hypo-fractionated stereotactic radiation therapy (SRT). WBRT can decrease the risk of distant brain metastasis, but it is associated with side effects such as cognitive dysfunction and fatigue that may reduce quality of life^5,6^. Stereotactic radiotherapy, including SRS and SRT, is often used to treat patients with limited number of brain metastases^7^. SRT is frequently administrated for larger tumours. Nevertheless, about 20% of brain metastases progress locally after stereotactic radiotherapy^8,9^. A priori prediction of local failure outcome for metastatic brain tumours treated with radiotherapy could facilitate early therapy adjustments or salvage treatments that are anticipated to improve survival and quality of life of patients.

Over the past decades, medical imaging has advanced in four distinct aspects including medical devices (hardware), imaging agents, standardized protocols for quantitative imaging and digital image analysis^10^. Radiomics focuses on improvements in automated quantitative image analysis. It includes systematic procedures to derive, organize and mine high-dimensional data from medical images for hypothesis generation/testing and improved decision support^11,12^. Several studies have demonstrated that quantitative imaging with radiomics has diagnostic and prognostic value in oncology with potential to increase precision in cancer management^13–22^. A number of previous studies have integrated quantitative imaging and genomic data analysis for further biological interpretation or better patient stratification in precision oncology^23–30^. Such parallel analyses have revealed important links between radiomic features and tumour genetics^31–34^.

The standard imaging modality to detect brain metastasis is magnetic resonance imaging (MRI) which plays a crucial role in brain tumour management. Karami et al*.* have recently developed a radiomic framework to predict the local control/local failure (LC/LF) outcome in brain metastasis within three months after SRT^35^. The quantitative MRI features were derived from gadolinium-contrast-enhanced T1-weighted (CE-T1w) and T2-weighted-fluid-attenuation-inversion-recovery (T2-FLAIR) images acquired before and at the first follow-up after SRT. The framework could predict the therapy outcome with a cross-validated sensitivity and specificity of 81% and 79%, respectively. Mouraviev et al*.* investigated whether MRI radiomic features in small brain metastases (median tumour volume of 0.12 cm^3^) complement standard clinical variables in predicting LC after SRS^36^.They developed predictive random forest models using MRI radiomic features and/or clinical variables. A total of 440 features were derived from the tumour core and the peri-tumoural regions, using the CE-T1w and T2-FLAIR images acquired at pre-treatment. Their model with selected clinical variables could predict LC outcome with a mean area under the receiver operating characteristic (ROC) curve (AUC) of 0.67. They indicated that adding optimized MRI radiomic features to the clinical variables can result in 19% relative increase in resampled AUC. A recent study explored the efficacy of quantitative computed tomography (CT) biomarkers derived from treatment-planning CT to predict local failure in brain metastasis treated with radiotherapy^37^. The predictive model developed in that study could predict the LC/LF outcome with an accuracy of 71% on an independent test set.

This study explored the efficacy of quantitative MRI coupled with machine learning techniques in a priori prediction of the LC/LF outcome in brain metastasis treated with hypo-fractionated SRT. Several predictive models were developed using the quantitative MRI and/or standard clinical features acquired at pre-treatment from 100 patients with brain metastases. The performance of the optimal models with quantitative MRI and clinical features were evaluated on an independent test set and compared. Further, the potential of quantitative MRI features in providing complementary information to improve the performance of clinical predictive models was investigated. Finally, the efficacy of the developed predictive models in differentiating patients in terms of survival was investigated and compared with those based on clinical criteria at post-treatment.

## Materials and methods

### Study protocol and data acquisition

**Figure 1:**
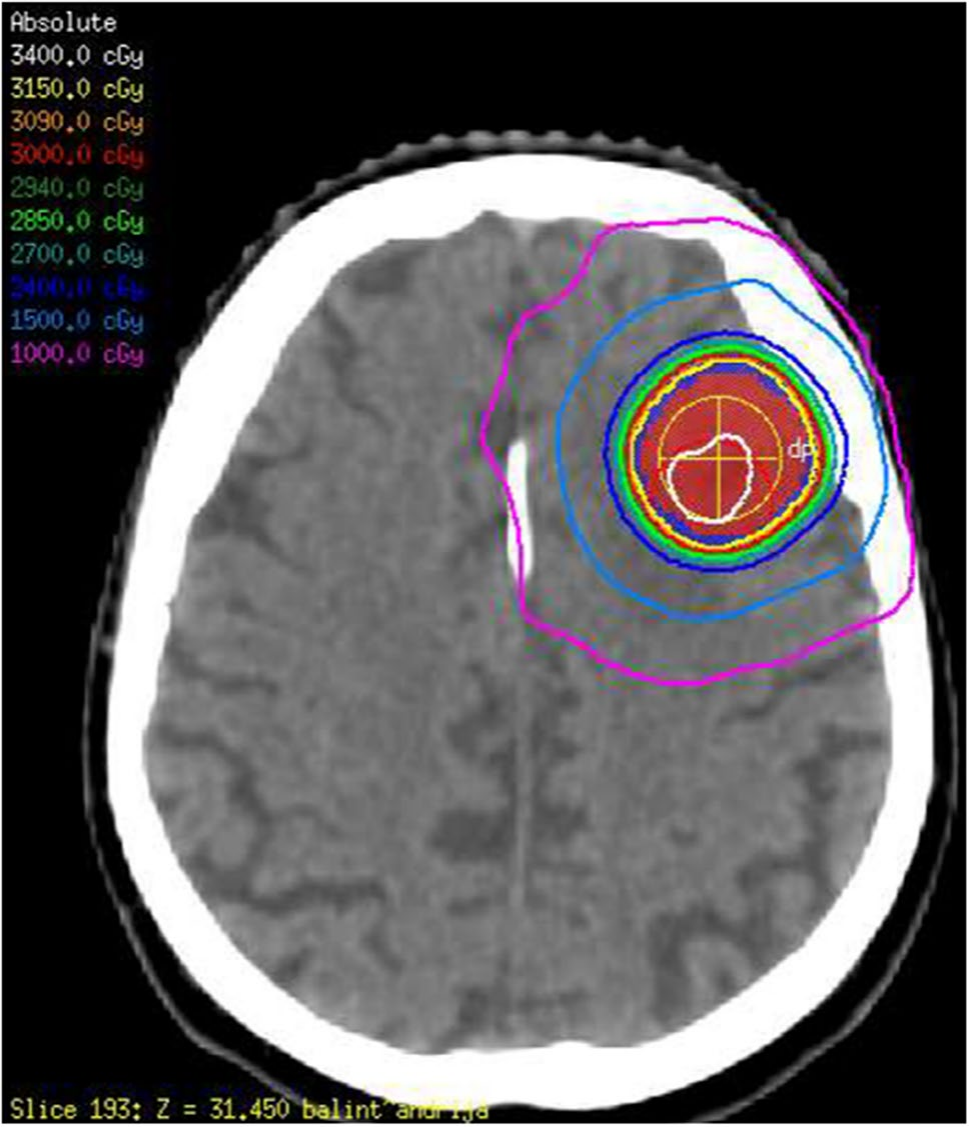
Treatment planning CT with the radiation isodose lines for a representative patient treated with 30 Gy in five fractions to a 2.7 cm frontal metastasis.

This study was conducted under the guidelines and regulations in accordance with institutional research ethics board approval from Sunnybrook Health Sciences Centre (SHSC), Toronto Canada. Imaging and clinical data were retrospectively acquired from 120 brain metastasis patients (171 lesions) treated with hypo-fractionated SRT. The Sunnybrook research ethics board granted a permission to use the retrospective data in the study without individual consent. Out of the 120 patients, 82 patients had a single target lesions for SRT, whereas 38 patients had multiple target lesions. All patients were treated on a linear accelerator (LINAC) with 22.5–35 Gy of radiation dose over five fractions, depending on size of the tumour, location in the brain and whether there was any prior radiotherapy. The treatment protocol was uniform and standardized within the institution, with a similar heterogeneity of dose in the target, prescribed at similar isodose^38,39^. Figure 1 presents an example of stereotactic radiotherapy plan with radiation isodose lines for a representative patient. CE-T1w and T2-FLAIR images were acquired for treatment-planning, and at follow-ups after the SRT on a 2–3 month schedule (up to six years) using a Philips 1.5 T Ingenia system (Best, Netherlands). The in-plane image resolution was 0.5 mm for both CE-T1w and T2-FLAIR images. The slice thickness was 1.5 mm and 5 mm for CE-T1w and T2-FLAIR images, respectively. The lesions were monitored longitudinally at follow-ups and the LC/LF outcome for each lesion was determined by a radiation oncologist and neuroradiologist using the serial imaging data. The LC/LF was defined as the outcome identified in the last patient follow-up. The RANO-BM criteria were used to determine an outcome of LF (progressive disease) or LC (complete response, partial response, or stable disease) for each lesion^6^. Local progression was differentiated from adverse radiation effect (ARE) based on the report by Sneed et al.^40^. All cases of ARE were diagnosed based on serial imaging (including the use of perfusion MRI), and/or histological confirmation^41^. Out of the 171 lesions, 108 lesions had an LC outcome whereas 63 lesions had LF after SRT (LF).

In this study, the performance of standard clinical variables in predicting treatment outcome was also investigated and compared with quantitative MRI features extracted from treatment-planning images. The clinical variables included histology, total dose of radiation (TD), number of brain metastases (NBM), location of tumour (supratentorium/infratentorium), maximum diameter of tumour (Max Diam), previous WBRT (yes/no), previous SRS or SRT (yes/no), and targeted systemic treatment (yes/no; for example, trastuzumab for HER2-positive breast cancer). Table 1 summarizes the patient characteristics.

**Table 1:** Patient characteristics and SRT outcome.

Data set	All	Training	Test
**Age**	Range: 21–92 years Mean: 63 years	Range: 21–92 Mean: 63	Range: 34–82 Mean: 64
**Maximum diameter of tumour (Max Diam)**	Range: 0.4–7 cm Mean: 2 cm	Range: 0.4–7 cm Mean: 1.9 cm	Range: 1–6 cm Mean: 2.2 cm
**Sex**			
Male	48 patients (40%)	44 Patients (44%)	4 Patients (20%)
Female	72 patients (60%)	56 Patients (56%)	16 Patients (80%)
**Number of brain metastases (NBM)**			
One lesion	42 patients (35%)	34 patients (34%)	8 patients (40%)
Two lesions	41 patients (34%)	37 patients (37%)	4 patients (20%)
Three or more lesions	37 patients (31%)	29 patients (29%)	8 patients (40%)
**Histology**			
Lung cancer	86 lesions (50%)	71 lesions (50%)	15 lesions (50%)
Breast cancer	41 lesions (24%)	33 lesions (23%)	8 lesions (27%)
Melanoma cancer	15 lesions (9%)	15 lesions (11%)	0 lesion (0%)
Colorectal cancer	9 lesions (5%)	7 lesions (5%)	2 lesions (7%)
RCC cancer	9 lesions (5%)	5 lesions (4%)	4 lesions (13%)
Other	11 lesions (7%)	10 lesions (7%)	1 lesion (3%)
**Location of tumour**			
Supratentorium	128 lesions (75%)	107 lesions (76%)	21 lesions (70%)
Infratentorium	43 lesions (25%)	34 lesions (24%)	9 lesions (30%)
**Previous WBRT**			
Yes	61 lesions (36%)	51 lesions (36%)	10 lesions (33%)
No	110 lesions (64%)	90 lesions (64%)	20 lesions (67%)
**Previous SRS or SRT**			
Yes	1 lesion (1%)	1 lesion (1%)	0 lesions (0%)
No	170 lesions (99%)	140 lesions (99%)	30 lesions (100%)
**Total dose of radiation (TD) in SRT (over 5 fractions)**			
22.5 Gy	1 lesion (1%)	1 lesion (1%)	0 lesion (0%)
25 Gy	29 lesions (17%)	23 lesions (16%)	6 lesions (20%)
27.5 Gy	8 lesions (5%)	6 lesions (4%)	2 lesions (7%)
30 Gy	104 lesions (60%)	87 lesions (62%)	17 lesions (57%)
32.5 Gy	13 lesions (8%)	9 lesions (6%)	4 lesions (13)
35 Gy	16 lesions (9%)	15 lesions (11%)	1 lesion (3%)
**Targeted systemic treatment**			
Yes	54 (32%)	43 (30%)	11 lesions (37%)
No	117 (68%)	98 (70%)	19 lesions (63%)
**Outcome of SRT**			
Crude LC	108 lesions (63%)	91 lesions (65%)	17 lesions (57%)
Crude LF	63 lesions (37%)	50 lesions (35%)	13 lesions (43%)

### Data pre-processing

**Figure 2:**
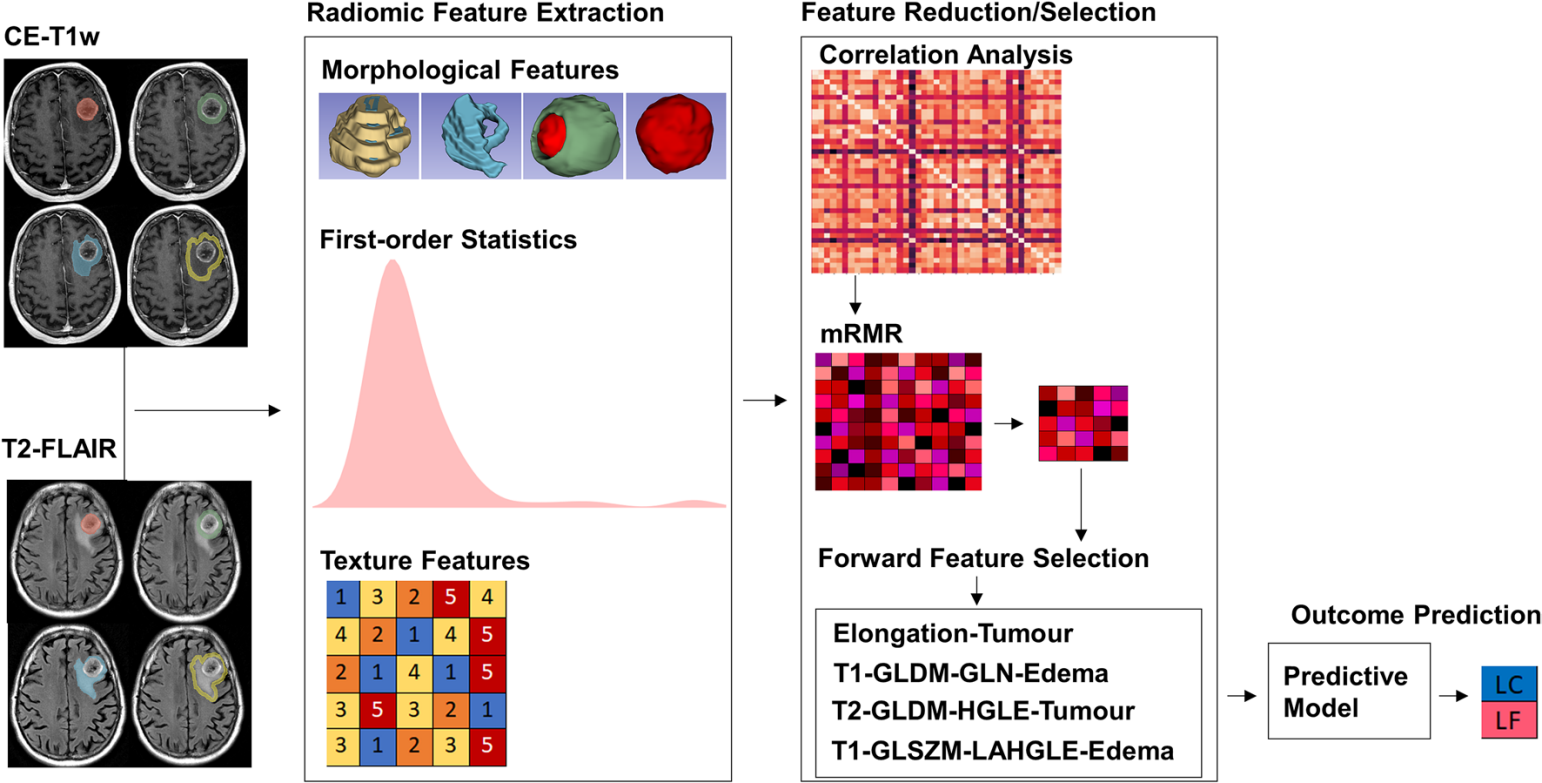
Scheme of the MRI radiomic framework for SRT outcome prediction.

The treatment-planning contours and CE-T1w and T2-FLAIR images were applied under supervision of an expert radiation oncologist to generate the tumour and edema masks for both images (Fig. 2). The tumour-margin and lesion-margin masks were generated for each lesion within the brain with up to 5 mm expansion around the tumour/lesion (tumour + edema) using morphological operations (the regions of the 5-mm margin lying outside the brain were removed from the mask). All images were resampled with a voxel size of $0.5\times 0.5 \times 0.5$ ${mm}^{3}$ to ensure a uniform scale in all directions when extracting the 3D features. The skull was stripped from the images using the skull stripper module in 3D slicer^42^. The image intensity was normalized within the brain in each image after skull stripping to have zero mean and unit variance.

The patients were partitioned into two independent sets of training (100 patients with 141 lesions) and test (20 patients with 30 lesions) using a stratified random sampling method^43^. The stratified random sampling method was used to have all parts of the data space represented similarly by both sets. The training set was applied for feature reduction/selection and to develop the outcome prediction models (described below), whereas the test set was used to evaluate the developed models independently on unseen cases.

### Feature extraction

Quantitative MRI features were derived from the tumour, edema, tumour-margin, and the lesion-margin areas using the Pyradiomics package in python^44^. A total of 800 features were extracted from the CE-T1w and T2-FLAIR images acquired at pre-treatment. The extracted features comprised morphological features, first-order statistics, and second-order texture features. The morphological features included least axis length, major axis length, flatness, elongation, minor axis length, maximum 3D diameter, maximum 2D diameter column, maximum 2D diameter row, maximum 2D diameter slice, mesh volume, surface area, sphericity, surface volume ratio, and voxel volume. The morphological features of tumour/tumour-margin and edema/lesion-margin were extracted from the corresponding region masks associated with the CE-T1w T2-FLAIR images, respectively. The first-order statistics including energy, uniformity, entropy, interquartile range, 10-percentile, 90-percentile, kurtosis, mean absolute deviation, robust mean absolute deviation, mean, median, maximum, minimum, range, skewness, root mean squared, total energy, and variance were extracted from the intensity histograms. The texture features were extracted based on four different statistical methods including gray-level co-occurrence matrix (GLCM), gray level dependence matrix (GLDM), neighborhood gray-tone difference matrix (NGTDM), and gray-level size-zone matrix (GLSZM). For texture analysis, the gray-level intensities in the region of interest (ROI) were quantized into bins with a fixed width of 25 such that the bin edges were equally spaced from zero. The scheme of the radiomic framework applied in this study has been presented in Fig. 2.

### Feature reduction and selection

A multi-phase feature reduction and selection process was adapted to construct the optimal quantitative MRI biomarkers for outcome prediction. The features were initially analyzed through a correlation-based analysis followed by a ranking process (described below) to reduce the number of redundant features for the feature selection procedure. A Pearson correlation analysis was applied to estimate the coefficient of determination (R^2^) for each feature pair. From each set of highly correlated features (${R}^{2}$ > 0.8) the feature with the highest prediction performance on the training set was retained as the representative feature and the other features were eliminated. The correlation-based feature reduction decreased the number of features from 800 to 192. These features were subsequently ranked using the minimal-redundancy-maximal-relevance (mRMR) criterion^45^, and the first 100 features in the list (as an upper-bound set of the appropriate features) were used in the feature selection procedure^46^. A sequential forward feature selection methodology was adapted to obtain the best feature set (optimal biomarker). To address the data imbalance issue during the feature selection, the majority class (LC) in the training set was undersampled by taking 501 random samples (without replacement) with the same size of the minority class (LF). The samples of the minority class were combined and shuffled with each of the undersampled subsets from the majority group to generate the balanced training subsets. To evaluate performance of different feature sets for feature selection, a stratified k-fold (k = 5) cross-validation was used on patient-level over each balanced subset, and the average cross-validated score was obtained over the 501 subsets. The area under the curve (AUC) obtained from the receiver operating characteristic (ROC) analysis was applied as the score for feature selection. A similar feature selection procedure was applied for the clinical variable.

### Outcome prediction

A k-nearest neighbor (k-NN) model (k = 5) was utilized with the selected features for LC/LF outcome prediction. Efficacy of the nearest neighbor methods in medical applications has recently been highlighted and explained in terms of theory and practice by Chen and Shah^47^. Similar to the methodology applied in feature selection, 501 balanced training subsets were generated to train different prediction models. For each lesion in the test set, a max-voting over the 501 outcomes predicted by these models determined the final predicted outcome^48^.

### Survival analysis

The Kaplan–Meier survival curves were generated for the two patient cohorts in the independent test set with an LC or LF outcome^49^. Two independent analyses were performed using the outcomes identified at pre-treatment using the predictive model, and at post-treatment based on the RANO-BM criteria as described in Study protocol and data acquisition section (ground truth). A patient with at least one tumour with an LF outcome were categorized into the LF cohort. A log-rank test was used to assess for statistically significant differences between the survival curves of the two patient cohorts.

## Results

The feature reduction/selection framework selected four radiomic features derived from treatment-planning CE-T1w and T2-FLAIR images as the optimal quantitative MRI biomarker for predicting outcome. The selected features included tumour elongation (Elongation-Tumour), GLDM gray level none-uniformity of edema in CE-T1w image (T1-GLDM-GLN-Edema), GLDM high gray level emphasis of tumour in T2-FLAIR image (T2-GLDM-HGLE-Tumour), and GLSZM large area high gray level emphasis of edema in CE-T1w image (T1-GLSZM-LAHGLE-Edema). Among the four features in the developed quantitative MRI biomarker, one describes the tumour shape, one quantifies the intra-tumour heterogeneity, and two characterize the heterogeneity in peri-tumoural areas (edema). Applying a similar feature selection procedure on the clinical variables resulted in four selected features as the best clinical feature set for outcome prediction including previous WBRT, targeted systemic treatment, TD, and histology.

**Figure 3:**
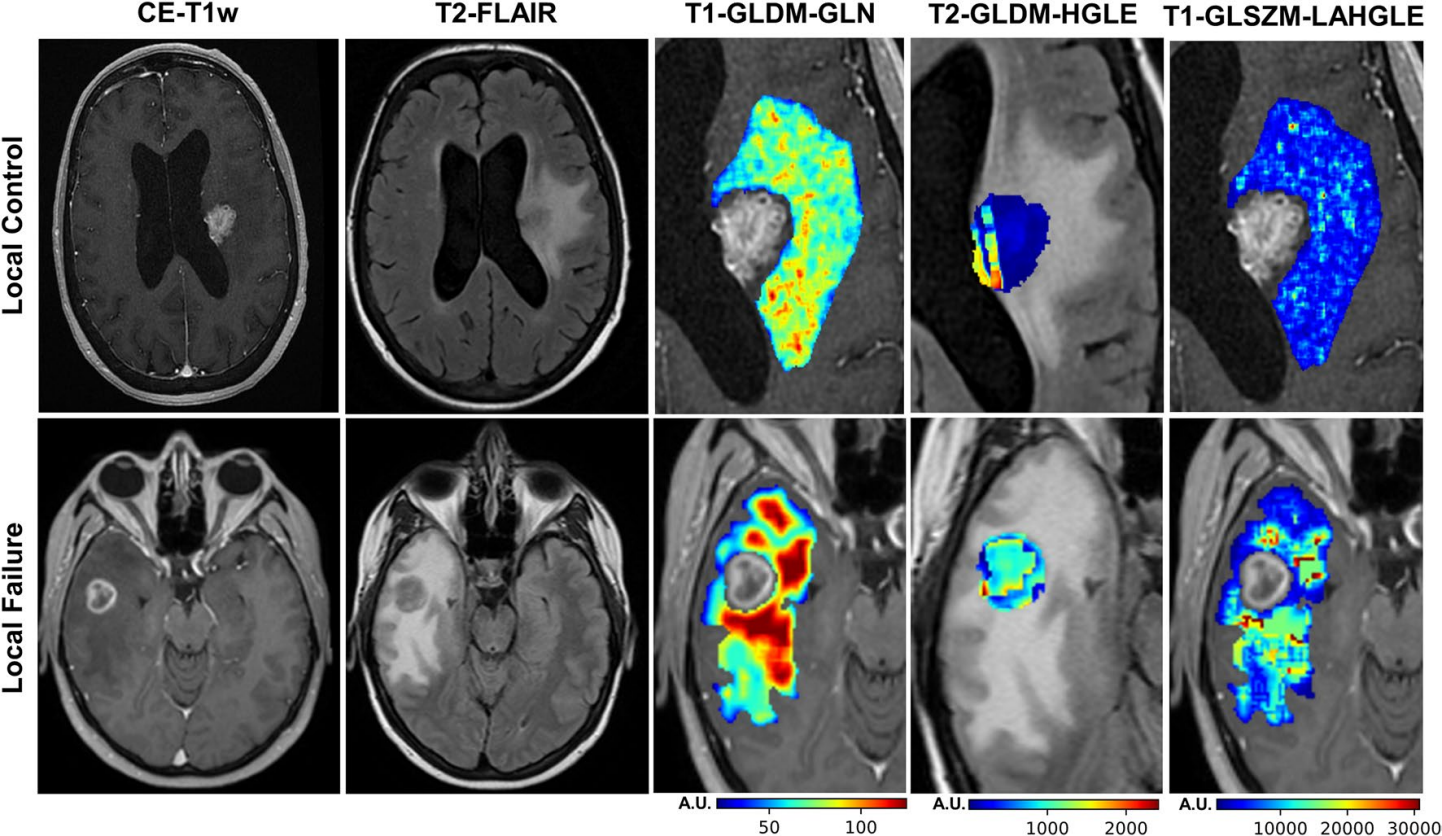
CE-T1w and T2-FLAIR images and parametric maps of the texture features in the optimal quantitative MRI biomarker for two representative lesions with LC and LF outcomes after SRT.

**Figure 4:**
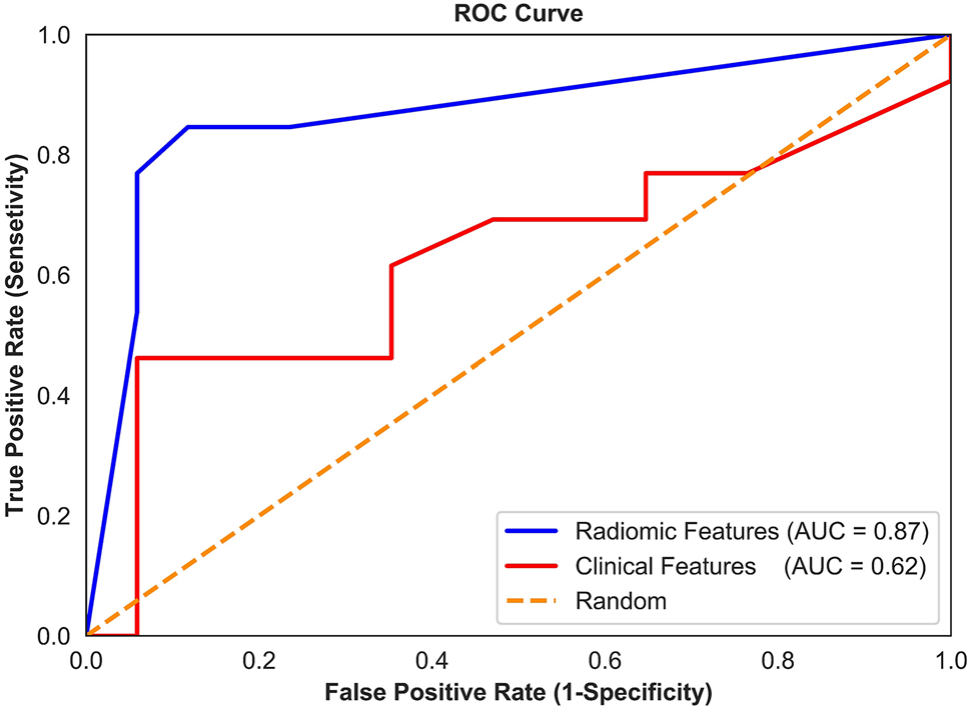
ROC curves associated with the outcome prediction models based on the radiomic and clinical features (presented in Table 2).

Figure 3 illustrates the pre-treatment CE-T1w and T2-FLAIR images and the parametric maps of the three texture features in the developed quantitative MRI biomarker for two representative lesions, one with an LC and the other with an LF outcome. The parametric maps demonstrate substantial differences between the two lesions in terms of spatial heterogeneity within the tumour and edema. Results of outcome prediction on the independent test set using the radiomic and clinical features have been presented in Table 2. The predictive model with the optimal quantitative MRI biomarker could predict the LC/LF outcome of lesions treated with SRT with a sensitivity, specificity, accuracy, and AUC of 88%, 85%, 87%, and 0.87, respectively. The model with the four selected clinical features (best clinical feature set) could predict the outcome with a sensitivity, specificity, accuracy, and AUC of 62%, 65%, 63%, and 0.62, respectively. Specificity (sensitivity) in this study refers to the ratio of the lesions having an LC (LF) outcome that was predicted with the correct outcome by the model. Figure 4 demonstrates the ROC curves associated with the outcome prediction models based on the best radiomic and clinical features, respectively.

**Table 2:** Results of a priori outcome prediction on the independent test set.

Features	Accuracy (%)	Sensitivity (%)	Specificity (%)	AUC
Radiomic Features: Elongation-Tumour, T1-GLDM-GLN-Edema, T2-GLDM-HGLE-Tumour, T1-GLSZM-LAHGLE-Edema	87	85	88	0.87
Clinical Features: Previous WBRT, Targeted Systemic Treatment, TD, Histology	63	62	65	0.62
Radiomic Features + Previous WBRT	77	69	82	0.76
Radiomic Features + (Previous WBRT, Targeted Systemic Treatment)	67	54	76	0.65
Radiomic Features + (Previous WBRT, Targeted Systemic Treatment, TD)	70	62	76	0.69
Radiomic Features + (Previous WBRT, Targeted Systemic Treatment, TD, Histology)	67	54	76	0.65
Clinical Features + Elongation-Tumour	70	69	71	0.70
Clinical Features + (Elongation-Tumour, T1-GLDM-GLN-Edema)	73	62	83	0.72
Clinical Features + (Elongation-Tumour T1-GLDM-GLN-Edema, T2-GLDM-HGLE-Tumour)	67	54	76	0.65
Clinical Features + (Elongation-Tumour, T1-GLDM-GLN-Edema, T2-GLDM-HGLE-Tumour, T1-GLSZM-LAHGLE-Edema)	67	54	76	0.65

The effects of adding clinical features to the radiomic features and vice versa for outcome prediction were investigated in a series of experiments. In the first set of experiments the selected radiomic features were added to the best clinical feature set incrementally, a new predictive model was developed, and the performance of the model was evaluated at each step on the independent test set. In the second set of experiments, the clinical features were added to the best radiomic feature set incrementally to develop new models. The results of outcome prediction with these models have been presented in Table 2. The results demonstrated that adding radiomic features to the clinical feature set could improve the AUC by up to 16% of its original value (0.72 versus 0.62). Particularly, incorporating the first radiomic feature (Elongation-Tumour) improved the AUC by 13%, and adding the second feature (T1-GLDM-GLNU-Edema) could improve the AUC by 16%. The results also demonstrated that adding clinical features to radiomic features does not necessarily result in an improvement in the performance of the predictive model. Incorporating the clinical features into the optimal quantitative MRI biomarker developed in this study decreased the performance of the predictive model relatively by 19% on average in terms of accuracy.

**Figure 5:**
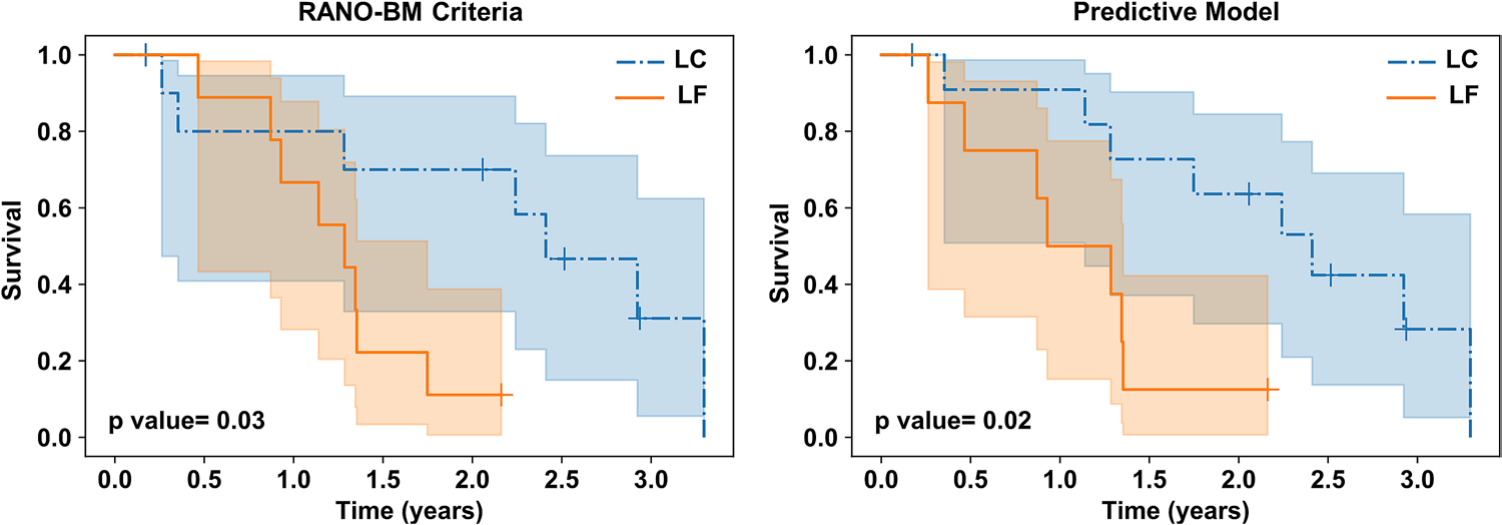
The survival curves of the patients treated with SRT and had lesions with LC versus LF outcome determined at the last patient follow-up based on the RANO-BM criteria (left), and at pre-treatment using the predictive model with the optimal quantitative MRI biomarker (right).

The performance of the optimal quantitative MRI biomarker was further evaluated on the independent test set through risk assessment in terms of survival analysis and compared with standard clinical criteria. Figure 5 shows the long-term survival curves of the patients in the LC and LF cohorts identified using the predictive model at pre-treatment, and based on the RANO-BM criteria at the last patient follow-up after SRT. The trends of the survival curves associated with the counterpart cohorts are similar in the two plots. The median survival in the cohorts identified based on the predictive model versus RANO-BM criteria was 26.2 versus 27.3 months for the LC, and 13.5 versus 15.6 months, for the LF. Both plots in Fig. 5 show a statistically significant difference (p-value < 0.05) in survival between the two patient populations (LC versus LF).

## Discussion and conclusion

This study investigated the potential of quantitative MRI radiomic features to develop an a priori predictive model for LC/LF outcome in metastatic brain tumours undergoing hypo-fractionated SRT. The radiomic features were derived from the treatment-planning CE-T1w and T2-FLAIR images to quantify morphology, signal intensity, and spatial heterogeneity of the tumour and peri-tumoural regions. The optimal quantitative MRI biomarker was constructed through a multi-phase feature reduction and selection procedure and includes features extracted from the tumour and edema regions. Results of outcome prediction on an independent test set demonstrated that the radiomics-based predictive model could classify the lesions at pre-treatment with a sensitivity, specificity, and accuracy of 85%, 88%, and 87% respectively. In comparison, the predictive model developed with the best clinical feature set classified the lesions with a sensitivity, specificity, and accuracy of 62%, 65%, and 63%, respectively.

The optimal quantitative MRI biomarker developed in this study consists of four features including Elongation-Tumour, T1-GLDM-GLN-Edema, T2-GLDM-HGLE-Tumour, and T1-GLSZM-LAHGLE-Edema. Elongation quantifies the relationship between the two largest principal components in the shape of an ROI and its value (inverse of true elongation) is in the range [0,1]. Objects with a line and circle shape have an elongation value of zero (lowest value) and one (highest value), respectively. A GLDM measures gray-level dependency in an ROI. The gray-level dependency is defined as the number of connected voxels within a specified neighbourhood with little difference in gray-level intensity compared to the center voxel. The GLDM gray-level none-uniformity (GLN) metric quantifies the similarity of gray-level intensity values in the ROI, where a lower GLDM-GLN value is associated with a greater similarity in intensity values. The GLDM high gray level emphasis (HGLE) quantifies the distribution of the higher gray-level intensity values, with a higher value indicating a greater concentration of high gray-level intensities in the ROI. A GLSZM quantifies the size of gray-level zones defined as connected voxels with the same gray-level intensity in an ROI. The GLSZM large area high gray level emphasis (LAHGLE) quantifies the joint distribution of larger size zones with higher gray-level values in the ROI. Out of the four selected radiomic features in this study, two features quantify heterogeneity within the edema, one feature characterizes intra-tumour heterogeneity, and one features describes the tumour morphology. The findings of this study confirm previous observations that heterogeneity in peri-tumoural areas is a crucial feature that should be quantified for therapy outcome prediction in brain metastasis^35,36^, and other malignancies^14,16^. Furthermore, the importance of tumour morphology features and especially tumour elongation for outcome prediction is in line with the observations in^36,37^. The texture features selected in this study for optimal biomarkers, were extracted from both T1w and T2-FLAIR images, with the second feature derived from T2-FLAIR. This implies the importance of both of these MRI sequences for outcome prediction in brain metastasis, and is in agreement with the findings of the previous studies^35,36,50^.

Our results demonstrated that incorporating radiomic features into the clinical predictive model could improve its overall performance in predicting LC/LF (16% relative improvement in AUC). Recently, Mouraviev et al*.* also reported a 19% relative increase in AUC for outcome prediction in brain metastasis undergoing SRS when radiomic and clinical features were applied together, compared to the clinical features alone^36^. The results of our study, however, demonstrated that adding clinical features to a radiomic-based outcome prediction model does not necessarily result in an improvement in its performance. Despite utilizing clinical features, such as histologic subtype, size of the tumour and radiation dose that have been shown to be predictive of survival of patients in several brain metastases studies, the radiomic features outperformed these factors^38,51,52^. One possible explanation is that clinical features of the relatively large tumours investigated in this study can partially be described by their morphological and textural features. In other words, these features are less predictive than the radiomic features applied in this study, and also do not complement the radiomic model. As such, forcing them into the model would only increase the dimension of the feature space, hence the complexity of problem, and can reduce the overall performance of the model.

The efficacy of the optimal quantitative MRI biomarker developed in this study was further assessed through long-term survival evaluations using the Kaplan–Meier analysis. The survival curves obtained for the patients with an a priori LC versus LF predicted outcome demonstrated a statistically significant difference. A similar difference was observed between the two patient cohorts identified many months later at post-treatment using the RANO-BM criteria. The results imply the potential of the outcome prediction models based on quantitative MRI to stratify the brain metastasis patients at pre-treatment into low and high risk groups with significantly different long-term outcomes, consistent with those based on standard clinical criteria that are available many months later after the treatment.

In conclusion, the results of this study on outcome prediction and survival assessment at pre-treatment are promising and demonstrate a good potential of the proposed methodology in improving clinical risk assessment and treatment planning for brain metastasis patients. However, for further assessment of the efficacy and robustness of the technique in the clinic and investigating its performance on smaller lesions and other dose/fraction regimens, subsequent studies are required on larger cohorts of patients and possibly with multi-institutional data.

## Data Availability

Data were collected and available at the Odette Cancer Centre, Sunnybrook Health Sciences Centre, Toronto, ON, Canada.
